# Diagnostic accuracy of multi-component spatial-temporal gait parameters in older adults with amnestic mild cognitive impairment

**DOI:** 10.3389/fnhum.2022.911607

**Published:** 2022-09-15

**Authors:** Shuyun Huang, Xiaobing Hou, Yajing Liu, Pan Shang, Jiali Luo, Zeping Lv, Weiping Zhang, Biqing Lin, Qiulan Huang, Shuai Tao, Yukai Wang, Chengguo Zhang, Lushi Chen, Suyue Pan, Haiqun Xie

**Affiliations:** ^1^Department of Neurology, First People’s Hospital of Foshan, Foshan, Guangdong, China; ^2^Department of Neurology, Nanfang Hospital, Southern Medical University, Guangzhou, Guangdong, China; ^3^National Research Center for Rehabilitation Technical Aids, Rehabilitation Hospital, Beijing, China; ^4^Dalian Key Laboratory of Smart Medical and Health, Dalian University, Dalian, China

**Keywords:** amnestic mild cognitive impairment, inertial-sensor gait analysis system, multi-kinematic parameters, diagnostic accuracy, receiver operating characteristic

## Abstract

**Objective:**

This study aimed to develop a diagnostic model of multi-kinematic parameters for patients with amnestic mild cognitive impairment (aMCI).

**Method:**

In this cross-sectional study, 94 older adults were included (33 cognitively normal, CN; and 61 aMCI). We conducted neuropsychological battery tests, such as global cognition and cognitive domains, and collected gait parameters by an inertial-sensor gait analysis system. Multivariable regression models were used to identify the potential diagnostic variables for aMCI. Receiver operating characteristic (ROC) curves were applied to assess the diagnostic accuracy of kinematic parameters in discriminating aMCI from healthy subjects.

**Results:**

Multivariable regression showed that multi-kinematic parameters were the potential diagnostic variables for aMCI. The multi-kinematic parameter model, developed using Timed Up and Go (TUG) time, stride length, toe-off/heel stride angles, one-leg standing (OLS) time, and braking force, showed areas under ROC (AUC), 0.96 [95% confidence interval (CI), 0.905–0.857]; sensitivity, 0.90; and specificity, 0.91. In contrast, a single kinematic parameter’s sensitivity was 0.26–0.95 and specificity was 0.21–0.90. Notably, the separating capacity of multi-kinematic parameters was highly similar to Montreal Cognitive Assessment (MoCA; AUC: 0.96 vs. 0.95). Compared to cognitive domain tests, the separating ability was comparable to Auditory Verbal Learning Test (AVLT) and Boston Naming Test (BNT; AUC: 0.96 vs. 0.97; AUC: 0.96 vs. 0.94).

**Conclusion:**

We developed one diagnostic model of multi-kinematic parameters for patients with aMCI in Foshan.

## Introduction

Alzheimer’s disease (AD), a neurodegenerative disease, is characterized by memory loss or multiple cognitive domain impairment. With rising longevity, AD is becoming a tremendous healthcare challenge worldwide. Although there have been many advances in pathogenesis and diagnosis of AD (Atri, 2019; Tiwari et al., 2019; Hameed et al., 2020). AD treatment is a challenge since there has been only symptomatic therapy available recently. Therefore, effective and accurate early screening of AD is crucial for intervention.

Alzheimer’s disease is a cognitive decline spectrum that includes preclinical and clinical stages. The disease course spans decades. Mild cognitive impairment (MCI) has been considered a prodromal stage of AD. It was divided into different subtypes depending on the impaired cognitive domains. Specifically, amnestic mild cognitive impairment (aMCI) was a transitional phase between normal aging and AD with a high AD conversion rate (Petersen, 2004). The familiar and widely used screening tools for aMCI are neuropsychological tests. The subjectivity and time consuming cannot be inadequate for current needs. Amyloid-42 of cerebrospinal fluid (CSF) and amyloid-positron emission tomography (PET) imaging has been increasingly accepted for the detection of aMCI (Hameed et al., 2020). However, given the invasiveness and high cost, CSF and PET imaging biomarkers were limited in clinical application (Guzman-Martinez et al., 2019). Therefore, more objective and convenient screening tools are necessary to identify aMCI accurately.

Recently, there has been a growing interest in motoric cognitive risk (MCR) syndrome, characterized as cognitive and motor dysfunction in older people (Verghese et al., 2013, 2014). Motor dysfunction is evaluated by multiple motion parameters usually, such as stride speed, stride length, stride time and variability, and so forth. Studies have found that motor and cognitive performance decline was paralleled in AD progression (Allali et al., 2016). In particular, stride speed was slow among individuals with early cognitive impairment (Knapstad et al., 2019), and stride length was shorter in patients with aMCI than in normal controls (Xie et al., 2019). Additionally, with the inertial-sensor gait analysis system integrated into applications, more spatial-temporal gait parameters were available (Prasanth et al., 2021).

There has been little research to investigate the diagnostic accuracy of gait parameters for identifying aMCI. Toosizadeh et al. (2019) applied objective tools for screening early AD and aMCI among oldest-old participants. The results showed that the cognitive status (both MCI and AD) was predicted with an area under the receiver operating characteristic (AUC) of 0.83 (sensitivity, 0.82; specificity, 0.72) (Toosizadeh et al., 2019). The task in the study was mainly based on elbow flexion with the dominant arm but not the gait parameters during walking. Thus, it was more suitable for the oldest-old or the elders with mobility impairments.

Additionally, several studies reported the diagnostic ability of motion parameters in MCI. Nielsen et al. (2018) suggested that the dual-task gait test, which was walking while simultaneously performing a cognitive challenge, had a high discriminative ability to separate patients with MCI from healthy controls (Nielsen et al., 2018). Another study evaluated MCI participants by a complex walking test. The results showed the sensitivity and specificity of motion parameters were 78 and 90% (Perrochon and Kemoun, 2014). The two studies showed that motion parameters had a high discriminative ability to separate MCI. However, MCI was a heterogeneous condition. It is necessary to focus on the aMCI subtype. In addition, motor functions were evaluated by either single parameter or complicated tasks in the two studies. The single parameter reflected a specific but not the overall motor function. In addition, the complex processes reduced the maneuverability in clinical practice. Hence, applying multi-kinematic parameters of relatively simple motor tasks might be preferable to distinguish aMCI from healthy elderly.

Therefore, this study investigated the diagnostic accuracy of multi-kinematic parameters collected by a sensor gait analysis system in simple gait tasks to distinguish aMCI.

## Methods

### Study design

This study is a cross-sectional study.

### Participants

**FIGURE 1:**
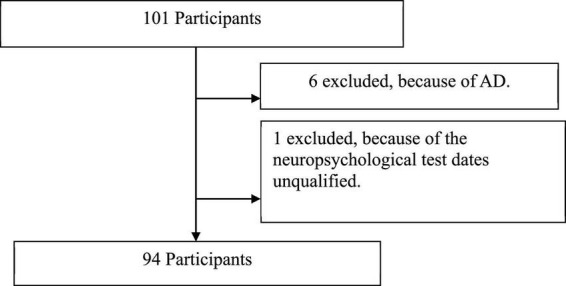
Flow diagram of participants.

From 101 participants initially recruited, we excluded seven cases because of the diagnosis of AD, and the neuropsychological test data were unqualified (Figure 1). Ninety-four older adults were included for analysis. Sixty-one participants were diagnosed with aMCI at neurological clinics of First People’s Hospital of Foshan between January 2020 and December 2020. Thirty-three cognitive normal (CN) older adults with matching demographic information (age, sex, and education level) were enrolled in the communities. Demographic characteristics, medical history, and Hamilton Depression Scale (HAMD) scores were collected during face-to-face interviews.

Inclusion criteria of aMCI were as follows (Petersen, 2004): (1) subjective cognitive complaint, preferably confirmed by an informant; (2) a single domain or multi-domain of memory decline. Abnormal objective cognitive domain impairment was identified by a cutoff of 1.5 SD, below education and age matched-specific norms; (3) preserved activities of daily living were confirmed by a clinician’s interviews; and (4) Global Clinical Dementia Rating (CDR) = 0.5.

Exclusion criteria for all participants were as follows: (1) illiteracy; (2) neurologic disorder and other systematic diseases that would likely contribute to cognitive and motor deficits (history of stroke, Parkinson’s disease, epilepsy, brain trauma, etc.), active rheumatic and orthopedic diseases that affect lower limbs, and history of knee/hip replacement; and (3) use of neuroleptics or benzodiazepines and psychiatric comorbidity (e.g., significant depressive/anxiety).

Ethics approval was obtained from the Research Ethics Board of the First People’s Hospital of Foshan. All participants signed written consent forms at the time of registration.

### Neuropsychological assessment

A neuropsychological test battery was carried out: global cognition was assessed using Montreal Cognitive Assessment (MoCA) (Julayanont et al., 2015) and CDR (Morris, 1993); memory with Auditory Verbal Learning Test (AVLT) that included immediate memory (AVLT_IM), short-delayed memory (AVLT_SDM, 5-min recalls), and long-delayed memory (AVLT_LDM, 20-min recalls) (Guo et al., 2001); language with Boston Naming Test (BNT); executive function with Stroop Color-Word Test (SCWT) that included part A, part B, and part C; attention with Symbol Digit Modalities Test (SDMT); and visual-spatial ability with Clock Drawing Test (CDT).

### Gait measurements

The gait data were collected by the JiBuEn^®^ gait analysis system (Tao et al., 2018; Gao et al., 2021). The system comprises wearable shoes and modules with the inertial Micro-Electro-Mechanical Systems (MEMS) sensors fixed under the shoe heel bottom, behind the upper and lower limbs, and wrist. The modules collected motion signals and transmitted them to a computer. The high-order low-pass filter and hexahedral calibration technique were employed in data preprocessing, which reduces high-frequency noise interference and installation errors produced by sensor devices. Moreover, the accumulative errors were also corrected based on the zero-correction algorithm. The final gait parameters were obtained by fusing acceleration data and posture, calculated using the quaternary complementary filtering technique.

Relative simple gait tasks, i.e., Free Walking, Timed Up and Go (TUG), and closed-eyed one-leg standing (OLS) tests, were implemented in this study. The gait parameters were collected during the gait tasks without cognitive challenge. Participants needed to walk on a walkway at their usual pace for the Free Walking test. The walkway was in a quiet, well-lit room. Participants wore specially made shoes walking for 30 s. The parameters of stride length, stride speed, cadence, stride time, toe-off/heel strike angles, and braking force came from the Free Walking test. TUG test measures in seconds, which was the time needed to rise from a chair, walk 3 m, turn around, and return to a seated position at a faster speed. Three times were tested for each subject. The mean time of three times was used for analysis. During the OLS test, participants were needed to stretch both arms to the sides, raise one leg about 5 cm from the floor, and stand up for as long as possible. The time of maintaining this position was measured. The subjects performed three attempts on each lower limb. The test ended when the subjects used the raised foot to touch the floor, moved the supporting leg on the ground, or observed a significant loss of balance. During the test, the examiner guarded the subject to prevent fell. The longest time of the three measurements was used for the analysis.

### Statistical analysis

The comparison of characteristics used a *t*-test or chi-square test between the two groups. Multivariable regression models were used to identify the potential diagnostic variables for aMCI. Age, sex, education, body mass index (BMI), hypertension history, and diabetes history were adjusted. Receiver operating characteristic (ROC) curves, constructed using bootstrap resampling (times = 500), were used to assess the diagnostic accuracy of multi-kinematic parameters in discriminating aMCI from healthy subjects. We drew ROC curves to plot the true positive values (sensitivity) against the false positive values (1-specificity) for different possible cutoff values of the respective markers. The ROC curves illustrate the ability of various parameters to classify healthy controls and aMCI correctly. The AUC values were calculated to measure the parameter’s overall accuracy.

Analyses were conducted by the statistical software packages R^1^ (the R Foundation) and Empower Stats^2^ (X&Y Solutions, Inc., Boston, MA, United States). All the *p*-values <0.05 were considered statistically significant.

## Results

### Participants characteristics

In total, 94 participants were included in the study, i.e., 61 elderly diagnosed with aMCI. Clinical dates are presented in Table 1—no significant group differences in demographic characteristics, visual-spatial ability, cadence, stride speed, and stride time. Compared to CN, participants with aMCI had poorer cognitive performance (such as global cognition, memory, language, executive function, and attention), longer TUG time, shorter stride length, smaller toe-off/heel stride angle, worse braking force, and OLS performance.

**TABLE 1:** Characteristics of the study participants.

	All (*n* = 94)	NC (*n* = 33)	aMCI (*n* = 61)	*P*-value^§^
Demographic characteristics				
Age, y	67.22 ± 4.36	68.03 ± 3.96	66.79 ± 4.53	0.188
Sex (male), *n*%	44 (46.81)	14 (42.42)	30 (49.18)	0.531
Education, *n*%				0.503^▲^
Primary school	26 (27.66)	8 (24.24)	18 (29.51)	
Secondary school	30 (31.91)	9 (27.27)	21 (34.43)	
Above secondary school	38 (40.43)	16 (48.48)	22 (36.07)	
BMI, kg/m^2^	23.19 ± 2.70	22.94 ± 2.69	23.32 ± 2.71	0.526
Hypertension history, yes, *n*%	34 (36.17)	15 (45.45)	19 (31.15)	0.168^▲^
Diabetes history, yes, *n*%	8 (8.51)	5 (15.15)	3 (4.92)	0.090^▲^
Neuropsychological assessment				
**MoCA, scores**	**24.37 ± 3.80**	**26.95 ± 2.08**	**22.80 ± 3.26**	**< 0.001**
**AVLT_immediate recall**	**4.49 ± 1.90**	**5.76 ± 1.62**	**3.80 ± 1.68**	**< 0.001**
**AVLT-SD (5-min delayed recall)**	**5.35 ± 2.61**	**7.33 ± 1.57**	**4.27 ± 2.43**	**< 0.001**
**AVLT-LD (20-min delayed recall)**	**4.30 ± 2.90**	**6.58 ± 1.94**	**3.05 ± 2.56**	**< 0.001**
CDT	8.47 ± 2.07	9.00 ± 1.48	8.18 ± 2.30	0.069
SCWT-A	12.33 ± 9.06	12.09 ± 14.13	12.46 ± 4.30	0.853
**SCWT-B**	**18.55 ± 6.97**	**16.03 ± 6.44**	**19.97 ± 6.90**	**0.009**
**SCWT-C**	**37.76 ± 18.25**	**32.76 ± 15.21**	**40.56 ± 19.31**	**0.049**
**BNT**	**20.91 ± 4.09**	**23.58 ± 3.00**	**19.45 ± 3.89**	**< 0.001**
**SDMT**	**34.66 ± 10.71**	**39.82 ± 6.60**	**31.67 ± 11.52**	**< 0.001**
Kinematics parameters				
**TUG time, s**	**15.26 ± 2.76**	**14.32 ± 2.36**	**15.76 ± 2.85**	**0.015**
**Stride length, cm**				
**Left**	**110.51 ± 15.80**	**115.85 ± 13.53**	**107.62 ± 16.28**	**0.014**
Right	110.46 ± 14.96	111.70 ± 11.15	109.79 ± 16.71	0.557
Stride speed, m/s				
Left	0.88 ± 0.15	0.90 ± 0.14	0.87 ± 0.16	0.326
Right	0.88 ± 0.15	0.90 ± 0.14	0.87 ± 0.16	0.326
Cadence, steps/min				
Left	95.89 ± 11.02	95.32 ± 10.78	96.20 ± 11.23	0.713
Right	95.89 ± 11.02	95.32 ± 10.78	96.20 ± 11.23	0.713
Stride time, s				
Left	1.29 ± 0.17	1.31 ± 0.19	1.27 ± 0.16	0.351
Right	1.25 ± 0.16	1.24 ± 0.14	1.25 ± 0.16	0.715
**Toe stride angle, degrees**				
Left	41.36 ± 4.64	42.47 ± 4.05	40.76 ± 4.86	0.089
**Right**	**41.17 ± 4.20**	**42.75 ± 3.63**	**40.31 ± 4.26**	**0.006**
**Heel stride angle, degrees**				
**Left**	**28.59 ± 9.47**	**31.46 ± 4.61**	**27.03 ± 10.99**	**0.030**
Right	28.42 ± 9.29	30.89 ± 4.22	27.08 ± 10.92	0.057
**Braking force**	**0.82 ± 0.10**	**0.86 ± 0.08**	**0.79 ± 0.10**	**< 0.001**
**One-leg standing test, s**				
Right	6.04 ± 8.06	7.45 ± 12.32	5.27 ± 4.15	0.212
**Left**	**6.32 ± 9.22**	**9.18 ± 13.08**	**4.75 ± 5.71**	**0.026**

BMI, body mass index; MoCA, Montreal Cognitive Assessment-Basic; AVLT, Auditory Verbal Learning Test; BNT, Boston Naming Test; SDMT, Symbol Digit Modalities Test; CDT, Clock Drawing Test; SCWT, Stroop Color-Word Test. Values of *p* < 0.05 are bolded. Bold font indicates they have statistical significance. ^§^Comparison based on unpaired *t*-test or chi-square test (^▲^).

### Multivariable regression of kinematic parameters and amnestic mild cognitive impairment

Table 2 shows that the potential diagnostic kinematic parameters for aMCI are TUG time, stride length, toe off/heel stride angle, OLS time, and braking force [TUG time, odds ratio (OR) = 1.31, 95% confidence interval (CI) = 1.04, 1.66; left stride length, OR = 0.95, 95% CI = 0.92, 0.99; right toe off stride angle, OR = 0.79, 95% CI = 0.66, 0.94; left heel stride angle, OR = 0.79, 95% CI = 0.68, 0.93; right heel stride angle, OR = 0.80, 95% CI = 0.69, 0.94; left leg standing time, OR = 0.93, 95% CI = 0.86, 1.00; and braking force, OR = 0.87, 95% CI = 0.80, 0.94].

**TABLE 2:** Regression analyses for kinematics parameters and amnestic mild cognitive impairment (aMCI).

	Crude	Model I	Model II
	
	OR (95%CI) P		
**TUG time**	**1.23 (1.04, 1.47) 0.018**	**1.31 (1.04, 1.65) 0.020**	**1.31 (1.04, 1.66) 0.024**
**Left stride length**	**0.97 (0.94, 0.99) 0.018**	**0.96 (0.92, 0.99) 0.019**	**0.95 (0.92, 0.99) 0.019**
Right stride length	0.99 (0.96, 1.02) 0.553	0.99 (0.95, 1.02) 0.447	0.98 (0.94, 1.02) 0.396
Left stride speed	0.24 (0.01, 4.05) 0.323	0.21 (0.01, 6.51) 0.375	0.19 (0.01, 6.49) 0.357
Right stride speed	0.24 (0.01, 4.05) 0.323	0.21 (0.01, 6.51) 0.375	0.19 (0.01, 6.49) 0.357
Left cadence	1.01 (0.97, 1.05) 0.709	1.02 (0.97, 1.07) 0.553	1.02 (0.96, 1.07) 0.569
Right cadence	1.01 (0.97, 1.05) 0.709	1.02 (0.97, 1.07) 0.553	1.02 (0.96, 1.07) 0.569
Left stride time	0.31 (0.03, 3.62) 0.348	0.20 (0.01, 3.98) 0.291	0.20 (0.01, 4.88) 0.324
Right stride time	1.68 (0.11, 26.82) 0.712	1.45 (0.03, 68.38) 0.849	1.13 (0.02, 57.81) 0.952
Left toe off angle	0.92 (0.83, 1.01) 0.092	0.90 (0.78, 1.03) 0.109	0.89 (0.77, 1.03) 0.113
**Right toe off angle**	**0.86 (0.76, 0.96) 0.009**	**0.82 (0.69, 0.96) 0.015**	**0.79 (0.66, 0.94) 0.007**
**Left heel stride angle**	**0.88 (0.80, 0.98) 0.014**	**0.83 (0.73, 0.95) 0.007**	**0.79 (0.68, 0.93) 0.003**
**Right heel stride angle**	**0.90 (0.82, 1.00) 0.044**	**0.82 (0.71, 0.95) 0.007**	**0.80 (0.69, 0.94) 0.006**
Right leg standing time	0.97 (0.91, 1.02) 0.242	0.97 (0.91, 1.04) 0.368	0.96 (0.90, 1.03) 0.254
**Left leg standing time**	**0.95 (0.89, 1.00) 0.053**	**0.95 (0.88, 1.01) 0.093**	**0.93 (0.86, 1.00) 0.047**
**Braking force**	**0.92 (0.87, 0.97) 0.001**	**0.88 (0.82, 0.95) 0.000**	**0.87 (0.80, 0.94) 0.000**

Model I adjust age, sex, and education; Model II adjusts age, sex, education, BMI, Hypertension history, and diabetes history. Bold font indicates they have statistical significance.

### Diagnostic accuracy of kinematic parameters for amnestic mild cognitive impairment

**FIGURE 2:**
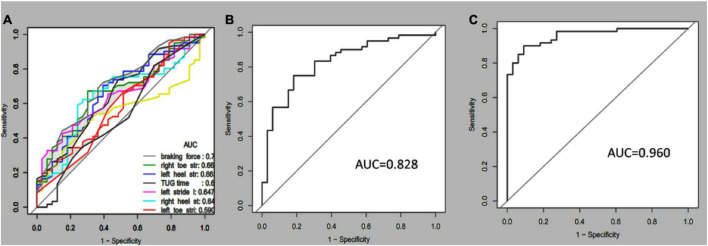
Receiver-operating characteristic (ROC) curves and corresponding area under the curve (AUC) of the single **(A)**, multi-kinematic **(B)**, parameters, and combined multi-kinematic parameters with age, sex, and education **(C)** to separate amnestic mild cognitive impairment (aMCI) from healthy controls.

Receiver operating characteristic curves were plotted to compare how gait tests differentiated aMCI from healthy people. Multi-kinematic parameters showed an AUC of 0.83, specificity of 0.82, sensitivity of 0.75, and accuracy of 0.77. When age, sex, and education were combined, the multi-kinematic parameters showed an AUC of 0.96, specificity of 0.91, sensitivity of 0.90, and accuracy of 0.90. In contrast, one single kinematic parameter’s sensitivity and specificity were 0.26–0.95 and 0.21–0.90 (Figure 2 and Tables 3, 4).

**TABLE 3:** Diagnostic ability of single/multiple kinematic parameters in amnestic mild cognitive impairment (aMCI).

	AUC	95%CI	Specificity	Sensitivity	Accuracy
**Single parameter**					
TUG time	0.651	0.534–0.766	0.515	0.737	0.659
Left toe stride angle	0.590	0.471–0.766	0.909	0.262	0.489
Right toe stride angle	0.668	0.556–0.779	0.697	0.672	0.680
Left heel stride angle	0.663	0.541–0.778	0.606	0.704	0.670
Right heel stride angle	0.646	0.528–0.762	0.727	0.623	0.659
Left stride length	0.646	0.535–0.758	0.848	0.426	0.574
Right stride length	0.544	0.427–0.660	0.667	0.525	0.574
Braking force	0.705	0.596–0.814	0.606	0.721	0.680
Left OLS time	0.584	0.460–0.706	0.212	0.950	0.688
Right OLS time	0.534	0.408–0.660	0.303	0.817	0.634
**Multiple parameters**	**0.828**	**0.741**–**0.915**	**0.818**	**0.750**	**0.774**

Bold values indicates they have statistical significance.

**TABLE 4:** Diagnostic accuracy of multi-kinematic parameters compared to neuropsychological tests in amnestic mild cognitive impairment (aMCI).

	AUC	95% CI	Specificity	Sensitivity	Accuracy	*P* ^§^
Model 1	0.758	0.659–0.857	0.878	0.606	0.702	–
**Model 1 + Multi-kinematic parameters**	**0.960**	**0.905–0.981**	**0.909**	**0.900**	**0.903**	–
Model 1 + MoCA	0.954	0.897–0.979	0.969	0.833	0.881	0.731
Model 1 + AVLT_SD	0.966	0.910–0.985	0.878	0.966	0.934	0.895
Model 1 + AVLT_LD	0.949	0.889–0.975	0.909	0.864	0.880	0.732
Model 1 + BNT	0.940	0.877–0.975	0.969	0.830	0.880	0.495
**Model 1 + SDMT**	**0.842**	**0.739**–**0.917**	**0.697**	**0.892**	**0.820**	**0.006**
**Model 1 + CDT**	**0.772**	**0.663**–**0.858**	**0.878**	**0.593**	**0.695**	**<0.001**
**Model 1 + SCWT_B**	**0.813**	**0.703**–**0.886**	**0.757**	**0.827**	**0.802**	**0.001**
**Model 1 + SCWT_C**	**0.800**	**0.689**–**0.874**	**0.818**	**0.724**	**0.758**	**<0.001**

Model 1 included age, sex, and education. Bold font indicates they have statistical significance. ^§^The *p*-value was the model compared to Model 1 + multi-kinematic parameters.

**FIGURE 3:**
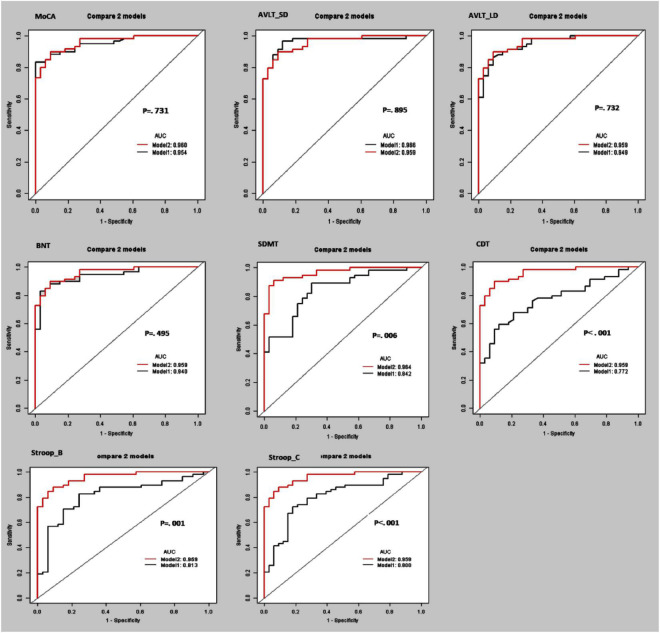
Receiver operating curve (ROC) curves and area under the ROC curve (AUC) of the multi-kinematic parameters (Model 2) when compared to neuropsychological tests (Model 1) to separate amnestic mild cognitive impairment (aMCI) from cognitive normal elderly.

Furthermore, the separating capacity of combined multi-kinematic parameters was highly similar to MoCA (AUC: 0.96 vs. 0.95; specificity: 0.91 vs. 0.97; sensitivity: 0.90 vs. 0.83; *p* = 0.731). Compared to cognitive domains tests, the separating capacity was comparable to AVLT (AVLT_SDM, AUC: 0.96 vs. 0.97; specificity: 0.91 vs. 0.88; sensitivity: 0.90 vs. 0.97; *p* = 0.895; AVLT_LDM, AUC: 0.96 vs. 0.95; specificity: 0.91 vs. 0.91; sensitivity: 0.90 vs. 0.86; *p* = 0.732) and BNT (AUC: 0.96 vs. 0.94; specificity: 0.91 vs. 0.97; sensitivity: 0.90 vs. 0.83; *p* = 0.495), superior to SDMT, CDT, and SCWT (Figure 3 and Table 4).

## Discussion

The present study developed one diagnostic model of multi-kinematic parameters for patients with aMCI. The model used TUG time, stride length, toe-off/heel stride angles, OLS time, and braking force. These findings have been observed for the first time in the elderly with aMCI.

Previous evidence suggested that motor function was disturbances among the elderly with aMCI. Studies reported that aMCI had slow stride speed (Ali et al., 2022), short stride length, and reduced gait stability (Xie et al., 2019). In addition, OLS performance was significantly worse in aMCI than in the CN subjects (Fujisawa et al., 2017). Furthermore, a few studies attempted to explore if gait tests can be an essential clinical detection tool for MCI. Åhman et al. (2020) suggested that the TUG test might be a useful complementary tool for diagnosing MCI. In another study, gait tests could distinguish MCI from depressive elderly (Metzger et al., 2016). Interestingly, the dominant arm elbow flexion test screened early AD and aMCI among the oldest-old participants. Results showed that the parameters could discriminate aMCI with sensitivity and specificity of 0.82 and 0.72 (AUC = 0.83) (Toosizadeh et al., 2019).

Given these studies, it tends to use gait parameters to screen for preclinical AD. However, the problem of selecting gait tests and kinematic parameters remains unsolved. The kinematic parameters showed a remarkable distinguishability for cognitive impairment, whether tested by TUG, motor-cognitive tasks, or arm elbow flexion. However, the multi-task TUG test was based on traditional tests. It is complex and labor-intensive. The kinematic parameters in the studies indicated certain aspects but not the comprehensive motor function. In addition, the participants were MCI elderly in most of the studies. MCI was a heterogeneous cognitive disorder. The conversion rate was 10–15% per year (Eshkoor et al., 2015). Thus, it is crucial to give more attention to aMCI, a subtype of the highest possible converted to AD.

With advancements in sensor technology, it has been possible to conveniently and automatically collect multiple temporal-spatial kinetic parameters. The inertial-sensor gait analytic system collected real-time and high-precision gait information by integrating electronic components, such as pressure, curvature, accelerometer, and gyroscope. Combined with an integrated angle meter module worn on limbs, the dynamic angle information can be obtained by blending it with foot information. Therefore, the inertial-sensor gait analytic system automatically provides comprehensive knowledge of individual gait (Tao et al., 2018; Prasanth et al., 2021).

This study investigated the diagnostic ability of multi-kinematic parameters by an inertial-sensor gait analytic system in aMCI. We obtained a diagnostic model using multi-kinematic parameters, with a sensitivity of 0.90, specificity of 0.91, and accuracy of 0.90 (AUC = 0.96). The multi-kinematic parameters included TUG time, toe-off/heel stride angle, stride length, braking force, and OLS time. Previous studies well-documented the significance of these kinematic parameters. TUG test assesses essential mobility skills and strength, balance, and agility (Bennell et al., 2011). It is reliable, safe, and time-efficient to evaluate overall functional mobility. Short stride length variability and high stride length variability predicted adverse clinical events in older adults (Bytyçi and Henein, 2021). Based on the inertial-sensor gait analysis system, the toe-off and heel stride angles were indicators of falling risk (Kanzler et al., 2015). Braking force was a gait marker associated with gait stability. It was weaker in patients with AD when compared to healthy elderly (Cheng et al., 2020). OLS test is one of the balance tests (Michikawa et al., 2009). Short standing time was associated with repeatedly falling in MCI elderly. Hence, this study improved convenience and automation by an inertial-sensor gait analytic system while considering comprehensive and sensitive parameters to reflect motion functions.

Furthermore, the separating capacity of multi-kinematic parameters was highly similar to MoCA; when compared to cognitive domains tests, the separating ability was comparable to AVLT and BNT. It is well-known that substantial memory impairment is the characteristic of aMCI (Albert et al., 2011; Jester et al., 2021). Additionally, language decline was one characteristic of aMCI independent of memory decline (Sherman et al., 2021). The shared neural substrate was the neuropathology mechanism of motor-cognition function decline. Hippocampus (Rosso et al., 2017), gray matter volume of frontal (Doi et al., 2017), and cortical thickness of bilateral superior temporal gyri (Ali et al., 2022) were associated with cognition and motor simultaneously. Thus, the results also verified the diagnostic accuracy of multi-kinematic parameters in distinguishing aMCI.

Overall, our results suggested that (1) the multi-kinematic parameters, such as TUG time, stride length, toe-off/heel stride angles, OLS time, and braking force, were potential diagnostic kinematic parameters for aMCI. (2) The model of multi-kinematic parameters helps doctors to initially identify the high risk of aMCI. These findings have significant implications for early, objective, and convenient discerning aMCI. Moreover, the inertial-sensor gait analytic system assessed the multi-kinematic parameters that might be accurate and suitable for dynamic motor and cognitive function monitoring.

The current study has some limitations that need to be considered. Firstly, this study is a cross-sectional design with its inherent deficiency. A follow-up examination is necessary for revealing the association between multi-kinematic parameters and aMCI in depth. Secondly, we did not classify aMCI due to the sample size limitation. More subjects are needed to analyze the value of multi-kinematic parameters in future screening and diagnosis of simple aMCI.

### Implications

This study has important clinical and practical implications for screening for early AD. The multi-kinematic parameters were automated and collected by an inertial-sensor-based gait analysis system. Specifically, the collections of the gait parameters were completed under simple gait tasks without cognitive challenge. If the findings are replicated in more extensive studies, they could represent a useful screening tool for early AD in clinical practice.

## Data availability statement

The raw data supporting the conclusions of this article will be made available by the authors, without undue reservation.

## Ethics statement

The studies involving human participants were reviewed and approved by the Research Ethics Board of the First People’s Hospital of Foshan. The patients/participants provided their written informed consent to participate in this study.

## Author contributions

SH, HX, SP, and XH contributed to the conception of the study and design, study supervision, interpretation of data, and manuscript preparation. CZ, YW, LC, and ST helped perform the analysis and critical revision of the manuscript. YL, PS, and JL contributed to acquiring kinetic parameters. WZ, QH, and BL contributed to purchasing neuropsychological evaluation. All authors contributed to the article and approved the submitted version.

## Footnotes

1
http://www.R-project.org

2
www.empowerstats.com
